# Chemical Profiling (HPLC-NMR & HPLC-MS), Isolation, and Identification of Bioactive Meroditerpenoids from the Southern Australian Marine Brown Alga *Sargassum paradoxum*

**DOI:** 10.3390/md13010102

**Published:** 2014-12-29

**Authors:** Robert Brkljača, Sylvia Urban

**Affiliations:** School of Applied Sciences (Discipline of Chemistry), Health Innovations Research Institute (HIRi) RMIT University, GPO Box 2476V Melbourne, Victoria 3001, Australia; E-Mail: robert.brkljaca@rmit.edu.au

**Keywords:** *Sargassum*, profiling, dereplication, HPLC-NMR, HPLC-MS, bioassay-guided, biological activity, meroditerpenoid

## Abstract

A phytochemical investigation of a southern Australian marine brown alga, *Sargassum paradoxum*, resulted in the isolation and identification of four new (**5**, **9**, **10**, and **15**) and nine previously reported (**1**, **2**, **6**–**8**, and **11**–**14**) bioactive meroditerpenoids. HPLC-NMR and HPLC-MS were central to the identification of a new unstable compound, sargahydroquinal (**9**), and pivotal in the deconvolution of eight (**1**, **2**, **5**–**7**, and **10**–**12**) other meroditerpenoids. In particular, the complete characterization and identification of the two main constituents (**1** and **2**) in the crude dichloromethane extract was achieved using stop-flow HPLC-NMR and HPLC-MS. This study resulted in the first acquisition of gHMBCAD NMR spectra in the stop-flow HPLC-NMR mode for a system solely equipped with a 60 μL HPLC-NMR flow cell without the use of a cold probe, microcoil, or any pre-concentration.

## 1. Introduction

Terpenoids and phenolic compounds represent the largest group of secondary metabolites reported from marine brown algae [1]. Marine brown algae belonging to the *Sargassum* genus (Sargassaceae, Fucales) number in excess of 300 species [2] located throughout most regions of the world [3,4], and are known to produce secondary metabolites such as meroterpenoids [1,5]. Meroditerpenoids are metabolites of mixed biogenesis containing terpenoid and non-terpenoid derived fragments. Meroditerpenoids derived from algae of the genus *Sargassum* are comprised of a polyprenyl chain attached to either a *p*-benzoquinone or hydroquinone moiety [6,7,8]. Variations of the meroditerpenoid structures occur mainly in the terpene side chain and include the addition of exocyclic double bonds, carboxylic acids, alcohols, or aldehyde functional groups [1,7,9]. Meroditerpenoids have been reported to display a range of biological activities including antibacterial, antioxidant, and antitumor activities [6,7,10,11,12].

**Figure 1:**
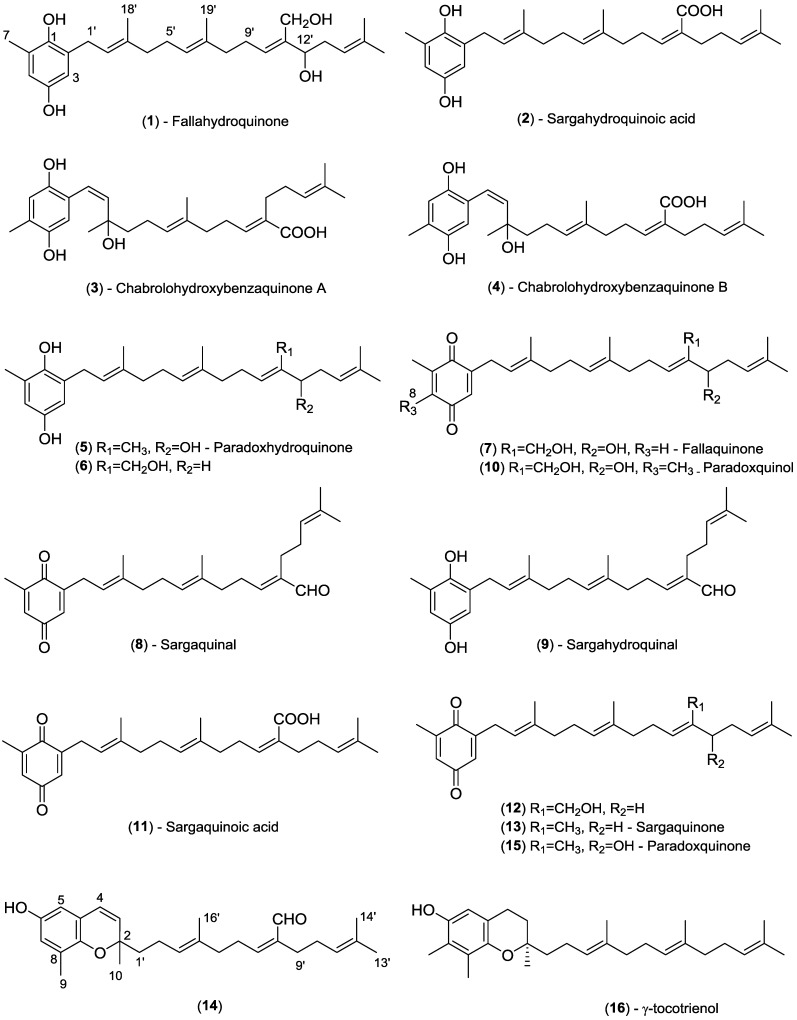
Structures of meroditerpenoids.

We recently examined a specimen of the marine brown alga *Sargassum paradoxum* (R. Brown ex Turner) Hooker and Harvey, collected from Port Phillip Bay, Victoria, Australia. This alga was selected for chemical investigation on the basis of the crude extract antimicrobial activity and that no previous chemistry had been reported from this particular *Sargassum* species [1,13]. Previous studies conducted by our research group on another closely related *Sargassum* species (*S. fallax*) resulted in the isolation of three new meroditerpenoids [7]. This also provided the motivation for studying this *Sargassum* species in that a comparison of the two species could potentially allow for chemotaxonomic markers to be identified. Herein we report the chemical profiling study conducted using HPLC-NMR and HPLC-MS together with the bioassay-guided isolation and structure determination of one new meroditerpenoid with a hydroquinone moiety paradoxhydroquinone (**5**) and two new meroditerpenoids possessing a *p*-benzoquinone moiety, paradoxquinol (**10**) and paradoxquinone (**15**). A new unstable meroditerpenoid, sargahydroquinal (**9**), was identified on the basis of the HPLC-NMR and HPLC-MS studies conducted. Revisions in the proton and/or carbon NMR chemical shift assignments for three of the meroditerpenoids (**1**, **2**, and **13**) have also been documented. In all, 14 meroditerpenoids (Figure 1) were detected in the crude extract of *S. paradoxum* and, of these, 13 could be identified using a combination of on-line (HPLC-NMR and HPLC-MS) and off-line (conventional isolation and structure elucidation) methodologies.

## 2. Results and Discussion

The frozen marine alga was extracted with 3:1 methanol/dichloromethane, evaporated under reduced pressure, and sequentially solvent partitioned (triturated) into dichloromethane- and methanol-soluble fractions, respectively. While both the dichloromethane- and methanol-soluble fractions displayed some antimicrobial activity, the dichloromethane extract was slightly more selective in its activity (Section 2.3). ^1^H NMR and analytical HPLC analyses indicated that the dichloromethane extract contained a series of structurally related compounds. On the basis of these observations, only the dichloromethane extract was further examined.

### 2.1. Chemical Profiling (HPLC-NMR & HPLC-MS)

**Figure 2:**
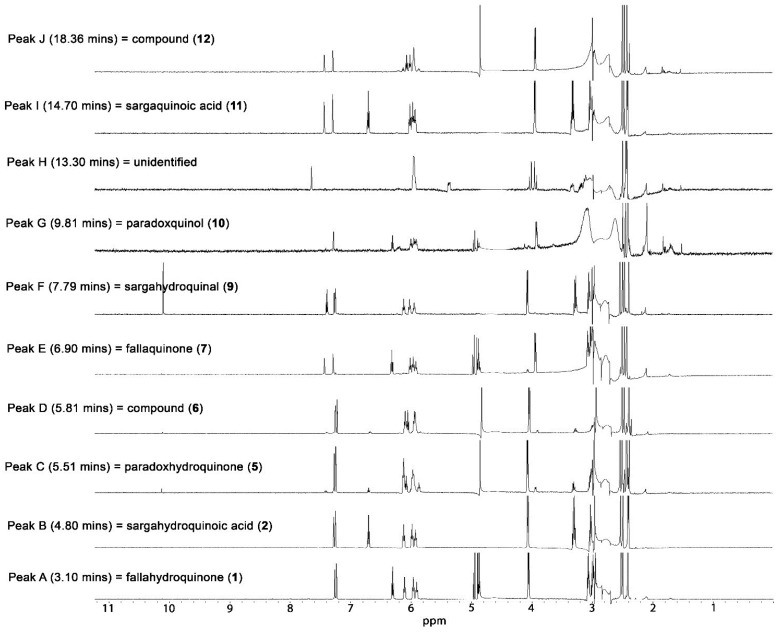
Stop-flow Wet1D proton NMR spectra (500 MHz, 75% CH_3_CN/D_2_O, suppression of HDO and CH_3_CN at t δ_H_ 4.64 and 2.82 ppm, respectively) of peaks A–J resulting from analysis of the dichloromethane crude extract of *S. paradoxum*.

The dichloromethane extract was subjected to both HPLC-NMR and HPLC-MS chemical profiling and a total of 10 peaks (A–J) were detected (Figure 2). Analysis of the stop-flow WET1D proton NMR spectra showed the presence of characteristic proton NMR signals in the aromatic and olefinic regions, together with various upfield proton NMR signals (Figure 2). Examination of the UV profile extracted for each of the peaks detected by PDA showed the presence of two distinct UV chromophores at λ_max_ 255 and 290 nm, typical of *p*-benzoquinones and hydroquinones that are known to occur in this genera of algae [1,13].

The two main peaks that were present in the dichloromethane crude extract of *S. paradoxum* (peaks A and B in Figure 2) were completely identified and characterized by HPLC-NMR and HPLC-MS. Both compounds displayed a UV maxima at 290 nm, supporting the presence of a hydroquinone moiety [14].

The HPLC-NMR stop-flow WET1D proton NMR spectrum of peak A (Figure 2) showed two *meta*-coupled protons at δ_H_ 7.22 and 7.25, indicating the presence of an aromatic moiety displaying an AB splitting pattern as well as deshielded proton signals at δ_H_ 4.95, 4.88, and 4.87. Complete analysis of the 1D and 2D NMR data (Table 1) obtained via stop-flow HPLC-NMR, and the high resolution ESI MS obtained for this peak via HPLC-MS analysis (*m*/*z* 427.2846 [M − H]^−^), allowed for the structure to be unequivocally elucidated as the previously described meroditerpenoid fallahydroquinone (**1**) [7].

The HPLC-NMR stop-flow WET1D proton NMR spectrum of peak B (Figure 2) supported the presence of a *meta*-coupled aromatic AB system (δ_H_ 7.23 and 7.26). Also present was a deshielded olefinic proton resonance (δ_H_ 6.69). The gHMBCAD NMR spectrum obtained in the stop-flow HPLC-NMR mode showed a signal at δ_C_ 170.9 ppm, confirming a carboxylic acid functional group (Table 1). Complete analysis of the 1D and 2D NMR data obtained via stop-flow HPLC-NMR and the high-resolution ESI MS obtained via HPLC-MS (*m*/*z* 425.2694 [M − H]^−^) suggested the previously described meroditerpenoid sargahydroquinoic acid (**2**) [8]. While a direct comparison of the proton NMR chemical shifts obtained via stop-flow HPLC-NMR to the NMR data reported in the literature is impeded by the expected chemical shifts differences that occur for spectra acquired using off-line conventional deuterated NMR solvents compared to HPLC-NMR solvents, the majority of the proton NMR signals for this compound varied from the literature data by a consistent factor. However, the proton chemical shift for position H-10′ (δ_H_ 6.69) differed to that reported for sargahydroquinoic acid (**2**) (δ_H_ 6.02) [8]. To confirm the 10′/11′ double bond configuration, single irradiation nOe NMR experiments were carried out whilst in the stop-flow HPLC-NMR mode. The proton at δ_H_ 6.69 was irradiated and a key nOe enhancement observed to the methylene protons at δ_H_ 3.02 (H-12′). This provided confirmation that peak B (Figure 2) has a 10′*Z* double bond configuration, which corresponds to the structure assigned to sargahydroquinoic acid. Further confirmation of the 10′*Z* double bond configuration came from comparison to the meroditerpenoids chabrolohydroxybenzaquinone A (**3**) and C (**4**), which were isolated as co-occurring double bond isomers from the soft coral *Nephthea charoli* [15]. These compounds show that there is a characteristic difference in the carbon chemical shift of the C-12’ methylene (δ_C_ 26.7 ppm and 34.5 ppm for the 10′*E* and 10′*Z* isomers, respectively) [15]. This γ effect is particularly valuable for distinguishing *E* and *Z* isomers of trisubstitued alkenes. Since the carbon for the C-12′ methylene in (**2**) displayed a carbon chemical shift at δ_C_ 35.3, this provided further confirmation for the 10′*Z* configuration.

**Table 1:** NMR data (500 MHz, 75% CH_3_CN/D_2_O, suppression of HDO and CH_3_CN at δ_H_ 4.64 and 2.82 ppm, respectively) for fallahydroquinone (**1**) and sargahydroquinoic acid (**2**) obtained via stop-flow HPLC-NMR.

	Fallahydroquinone (1)				Sargahydroquinoic Acid (2)								
Position	δ_C_^a^, Type	δ_H_ (*J* in Hz)	gCOSY	gHMBCAD	δ_C_^a^, Type	δ_H_ (*J* in Hz)		gCOSY	gHMBCAD			nOe	
1	146.0, C				146.0, C								
2	ND ^b^				130.4, C								
3	114.4, CH	7.22, d (3.0)			114.4, CH	7.23, d (3.0)		1’	1, 4, 5				
4	ND ^b^				150.6, C								
5	115.6, CH	7.25, d (3.0)	7		115.6, CH	7.26, d (3.0)		7	1, 3				
6	126.9, C				126.9, C								
7	ND ^b^	2.96, s	5	1, 5, 6	ND ^b^	2.96, s			1, 5, 6				
1′	29.3, CH_2_	4.05, d (7.5)	2′, 18′	1, 3, 2′, 3′	29.3, CH_2_	4.06, d (7.0)		3, 2′, 18′	1, 2, 3, 2′, 3′				
2′	123.4, CH	6.10, t (7.5)	1′, 18′		123.4, CH	6.10, t (7.0)		1′, 18′	1′, 4′, 18′				
3′	137.0, C				137.1, C								
4′	40.2, CH_2_	SS ^c^			40.2, CH_2_	2.91, m							
5′	ND ^b^	2.94, m			ND ^b^	2.94, m		6′, 19′	3′, 4′, 6′, 7′				
6′	125.4, CH	5.96, t (7.0)	5′, 19′		125.6, CH	5.97, t (7.0)		5′, 19′	8’, 19’				
7′	135.6, C				135.4, C								
8′	40.1, CH_2_	SS ^c^			39.6, CH_2_	2.91, m		9′					
9′	ND ^b^	3.01, m	10′	10′, 11′	28.4, CH_2_	3.30, dt (7.0, 7.5)		10′	7′, 8′, 10′, 11′				
10′	130.7, CH	6.30, t (7.0)	9′		142.6, CH	6.69, t (7.5)		9′	8′, 12′, 20′			9′, 12′	
11′	140.3, C				132.5, C								
12′	75.7, CH	4.87, t (7.5)	13′	10′, 11′	35.3, CH_2_	3.02, m			10′, 11′, 13′, 14′, 20′				
13′	35.4, CH_2_	3.06, dd (7.0,7.5)	12′, 14′, 16′, 17′	12′, 14′, 15′	28.3, CH_2_	2.91, m		17′					
14′	121.6, CH	5.90, dd (7.0)	13′		124.3, CH	5.91, t (7.0)		13′, 16′, 17′	16′				
15′	134.1, C				133.1, C								
16′	18.0, CH_3_	2.41, s		14′, 15′, 17′	17.7, CH_3_	2.39, s		14′	14′, 15′, 17′				
17′	25.9, CH_3_	2.49, s	13′	14′, 15′, 16′	25.7, CH_3_	2.49, s		14′	14′, 15′, 16′				
18′	16.2, CH_3_	2.52, s	1′	2′, 3′, 4′	16.2, CH_3_	2.53, s		1′, 2′	1′, 2′, 3′, 4′				
19′	16.1, CH_3_	2.41, s			15.9, CH_3_	2.41, s		6′	6′, 7′, 8′				
20a′	57.8, CH_2_	4.95, d (12.5)		10′	170.9, C								
20b′		4.88, d (12.0)											
1-OH		ND ^b^				ND ^b^							
4-OH		ND ^b^				ND ^b^							
12′-OH		ND ^b^											
20′-OH		ND ^b^				ND ^b^							

^a^ Carbon NMR assignments made on the basis of gHSQCAD and gHMBCAD NMR experiments; ^b^ Signal not detected due to signal suppression; ^c^ Signal suppressed.

The HPLC-NMR stop-flow WET1D proton NMR spectra of peaks C and D resembled that of peak A [fallahydroquinone (**1**)] (Figure 2), both of which eluted closely. Upon closer examination, it was evident that peak D was a meroditerpenoid that contained one alcohol functional group, due to the presence of a deshielded methylene proton at δ_H_ 4.84. Examination of the high resolution-ESI MS obtained via HPLC-MS for peaks C and D indicated that these were isobaric and that they both possessed a UV maxima at 290 nm, supportive of a hydroquinone moiety [14]. On the basis of the mass difference and the proton NMR differences it was proposed that peak D contained a terminal alcohol moiety but, unlike fallahydroquinone (**1**), did not contain a secondary alcohol functional group; this led to peak D being identified as the previously reported compound 2-[11-(hydroxymethyl)-3,7,15-trimethyl-2,6,10,14-hexadecatetraen-1-yl]-6-methyl-1,4-benzenediol (**6**) [16]. This represents the first report of **6** occurring as a natural product. Due to suppression of the HDO peak, no deshielded proton resonances could be observed for peak C; however, in consideration of the slight elution time difference, and the identical molecular masses, it was suspected that peak C contained an oxymethine group. While in stop-flow HPLC-NMR, the closely eluting compounds showed diffusion, and the oxymethine signal at δ_H_ 4.84 could clearly be distinguished via on-flow HPLC-NMR as being present in peak D and absent in peak C (see Supplementary Information file). The structure of peak C was proposed to be the new meroditerpenoid 2-[12-hydroxy-3,7,11,15-tetramethyl-2,6,10,14-hexadecatetraen-1-yl]-6-methyl-1,4-benzenediol, attributed the trivial name paradoxhydroquinone (**5**). Although subsequent off-line isolation of peaks C and D afforded an inseparable mixture, identification of these peaks was possible via the chemical profiling approach since individual WET1D proton and gCOSY NMR data could be obtained separately for each peak. This highlights an important feature of HPLC-NMR: typically co-eluting secondary metabolites that are difficult to obtain separately by conventional off-line isolation approaches can be obtained as separate entities via stop-flow HPLC-NMR.

Peak E was determined to be the previously described meroditerpenoid fallaquinone (**7**) [7], based on the observed UV maxima, together with the stop-flow WET1D proton NMR spectrum and the high-resolution ESI MS obtained via HPLC-MS, which showed the presence of an ion at *m*/*z* 425.2694 [M − H]^−^.

On the basis of the HPLC-NMR stop-flow WET1D proton NMR spectrum of peak F and the UV profile (λ_max_ 290 nm), this compound also possessed a hydroquinone moiety [14]. The high-resolution ESI MS ion *m*/*z* of 409.2745 [M − H]^−^ obtained via HPLC-MS suggested a molecular formula of C_27_H_38_O_3_. The stop-flow WET1D proton NMR spectrum showed a distinct proton signal at δ_H_ 10.07, s, confirming the presence of an aldehyde moiety. The MarinLit database indicated that sargaquinal (**8**), which possesses a *p*-benzoquinone moiety and an aldehyde functional group, had been previously reported from the *Sargassum* genus [17]. Peak F was proposed as being the new meroditerpenoid (possessing a hydroquinone moiety), attributed the name sargahydroquinal (**9**). During subsequent off-line bioassay-guided isolation, sargahydroquinal (**9**) oxidized into sargaquinal (**8**) before any off-line data could be obtained. Owing to the low abundance of sargahydroquinal (**9**) in the crude dichloromethane extract, only the stop-flow WET1D proton NMR and gCOSY data were obtained in the HPLC-NMR mode for sargahydroquinal (**9**) and are provided in Table 2. By comparison of the off-line data for sargaquinal (**8**) to peak F, the structure of sargahydroquinal (**9**) could be established. The configuration of the double bonds for sargaquinal (**8**) have been reported as 2′*E*, 6′*E*, and 10′*E* [17]. A bsNOESY NMR experiment carried out for sargaquinal (**8**) revealed nOe enhancements between the olefinic protons at positions H-2′ and H-6′ to the methylene protons at positions H-4′ and H-8′, along with the olefinic proton at position H-10′ showing a nOe enhancement to the aldehyde proton at position H-20′. On biosynthetic grounds, the same configuration was attributed to the double bonds in sargahydroquinal (**9**).

**Table 2:** NMR data (500 MHz, 75% CH_3_CN/D_2_O, suppression of HDO and CH_3_CN at t δ_H_ 4.64 and 2.82 ppm, respectively) for sargahydroquinal (**9**) obtained via stop-flow HPLC-NMR.

	Sargahydroquinal (9)	
Position	δ_H_ (*J* in Hz)	gCOSY
1		
2		
3	7.23, d (2.5)	
4		
5	7.25, d (2.5)	
6		
7	2.94, s	
1′	4.05, d (7.5)	2′, 18′
2′	6.10, t (7.5)	1′
3′		
4′	2.70-3.00, m	
5′	2.96, m	6′
6′	6.00, t (7.0)	5′
7′		
8′	2.98, m	9′
9′	3.26, dt (7.0, 8.0)	8′, 10′
10′	7.37, t (7.0)	9′
11′		
12′	2.70-3.00, m	
13′	2.70-3.00, m	
14′	5.92, t (7.0)	16′
15′		
16′	2.37, s	14′
17′	2.48, s	
18′	2.52, s	1′, 2′
19′	2.35, s	
20′	10.07, s	
1-OH	ND ^a^	
4-OH	ND ^a^	

^a^ Signal not detected.

The UV profile (λ_max_ 255 nm_)_ observed for peak G in the HPLC-NMR and HPLC-MS analyses confirmed a *p*-benzoquinone moiety [14]. The HPLC-NMR stop-flow WET1D proton NMR spectrum of peak G showed close resemblance to fallaquinone (**7**), except that an aromatic proton NMR resonance was absent. Comparison of the mass of peak G to fallaquinone (**7**), observed from ESI MS obtained via HPLC-MS analysis, showed a difference of 14 mass units, indicating that one of the aromatic proton signals had been replaced by a methyl substituent. The structure of peak G was therefore proposed to be the new meroditerpenoid 5-[12*R*-hydroxy-11-(hydroxymethyl)-3,7,15-trimethyl-2,6,10,14-hexadecatetraen-1-yl]-2,3-dimethyl-1,4-benzoquinone, attributed the trivial name paradoxquinol (**10**).

Peak I showed close similarities to sargahydroquinoic acid (**2**) and was identified as the previously reported sargahydroquinoic acid (**11**) [7,17], the benzoquinone counter part of **2**.

Peak J possessed a *p*-benzoquinone moiety from the UV chromophore at 263 nm [14]. Analysis of the HPLC-NMR stop-flow WET1D NMR data showed close resemblance to **6**. On the basis of the molecular ion deduced from the high-resolution ESI MS (409.2742 [M − H]^−^) obtained via HPLC-MS, in consultation with a SciFinder search, peak J was identified as previously described meroditerpenoid 2-[11-(hydroxymethyl)-3,7,15-trimethyl-2,6,10,14-hexadecatetraen-1-yl]-6-methyl-1,4-benzoquinone (**12**) [16,17]. Although subsequent off-line isolation afforded **12** and **15** as an inseparable mixture, identification of these compounds was possible via the chemical profiling approach since individual WET1D and gCOSY NMR data could be obtained separately for **12**, which could then be related back to the mixture.

### 2.2. Off-Line Bioassay-Guided Isolation

Following the chemical profiling (HPLC-NMR and HPLC-MS) conducted on the dichloromethane crude extract, a bioassay-guided isolation approach was undertaken to deduce the nature of the bioactive constituent(s) responsible for the crude extract bioactivity. The off-line isolation also allowed for any further identification and characterization of the meroditerpenoids concluded on the basis of the chemical profiling analysis as well as identifying any additional minor constituents not detected by HPLC-NMR.

A portion of the dichloromethane extract was fractionated by silica gel flash chromatography and selected fractions were evaluated for antimicrobial activity. Fractions for further purification were selected based on the observed antimicrobial activity, analysis of the ^1^H NMR spectra, and the analytical HPLC chromatograms. Purification was conducted using reversed phase HPLC resulting in the isolation of 12 meroditerpenoids, eight (**1**, **2**, **5**–**7**, and **10**–**12**) of which were previously detected by HPLC-NMR and HPLC-MS analyses, and four of which were additional meroditerpenoids (**8** and **13**–**15**) that were not observed in the chemical profiling. Nine known meroditerpenoids (**1**, **2**, **6**–**8**, and **11**–**14**) were isolated and identified by direct comparison of their NMR spectroscopic and mass spectrometry data with that reported in the literature [7,8,16,17,18,19]. The off-line study also confirmed the structures of **1**, **2**, **5**–**7**, and **9**–**12** as deduced by HPLC-NMR and HPLC-MS.

Fallahydroquinone (**1**) was isolated as an unstable yellow oil. Analysis of the 2D NMR data showed that the carbon NMR chemical shifts for positions C-2 and C-6, reported as occurring at δ_C_ 125.4 and 127.8, respectively, should be reassigned to δ_C_ 127.8 (C-2) and δ_C_ 125.4 (C-6), respectively. In addition, the proton NMR signals for the position H-4′ and H-8′ protons reported at δ_H_ 2.01 and 2.08, respectively, should also be reversed. The specific rotations of the previously reported meroditerpenoids fallahydroquinone (**1**) and fallaquinone (**7**) were compared to literature values [7]. While the specific rotation for fallahydroquinone (**1**) was comparable in sign and magnitude, the specific rotation of fallaquinone (**7**) differed to that reported. The purity of fallaquinone (**7**) in this study was higher than that obtained in the original isolation. It was concluded that the impurities present were sufficient to cause the specific rotation to vary in sign and magnitude. The accuracy of the specific rotations was considered given the low quantities isolated. The relevant compounds isolated in this study were of high purity, and did not display small specific rotations. That both the specific rotation of both fallahydroquinone (**1**) and fallaquinone (**7**) are of the same sign and magnitude is consistent with the fact that the former oxidizes to the latter with retention of configuration.

Sargahydroquinoic acid (**2**) was isolated as an unstable oil. Analysis of the 2D NMR data showed that the carbon NMR chemical shifts for positions C-9′ and C-13′, reported as occurring at δ_C_ 27.8 and 28.3, respectively, should be reassigned to δ_C_ 28.3 (C-9′) and δ_C_ 27.8 (C-13′), respectively.

Comparison of the 2D NMR data of sargaquinone (**13**) with the literature data indicated discrepancies with some of the carbon NMR chemical shift assignments. The carbon NMR chemical shifts for positions C-3, C-5, C-3′, and C-15′ were either incorrectly assigned or not assigned. These carbon NMR chemical shifts have been unequivocally established as occurring at δ_C_ 132.3, 133.2, 140.0 and 131.3, respectively.

Sargahydroquinal (**9**) and one other unidentified peak (peak H) (Figure 2) degraded during the off-line isolation, which meant that it was not possible to conduct a complete 2D NMR or antimicrobial activity evaluation on these constituents.

Paradoxhydroquinone (**5**) was isolated off-line as an inseparable mixture with 2-[11-(hydroxymethyl)-3,7,15-trimethyl-2,6,10,14-hexadecatetraen-1-yl]-6-methyl-1,4-benzenediol (**6**) in a ratio of approximately 3:5. While complete 2D NMR characterization was conducted on the mixture, these components were obtained as separate entities via stop-flow HPLC-NMR. By analysis of the data obtained from both chemical profiling and off-line isolation, the compounds could be identified. In the first instance, the previously reported compound 2-[11-(hydroxymethyl)-3,7,15-trimethyl-2,6,10,14-hexadecatetraen-1-yl]-6-methyl-1,4-benzenediol (**6**) could be confirmed [16]. The comparison of the aromatic proton signals of **5** with those of the known meroditerpenoids isolated supported the presence of a hydroquinone moiety. The structure of **5** as proposed by chemical profiling was confirmed by analysis of the gHMBCAD NMR spectrum, which indicated that the proton signal at δ_H_ 3.97 (H-12′) showed correlations with the carbons at δ_C_ 126.2 (C-10′), 120.2 (C-14′), and 11.7 (C-20′) (Table 3). The oxymethine group was concluded to be located at position 12′ on the basis of the HMBC NMR correlation observed from the singlet methyl group at position H-20′ (δ_H_ 1.61, s) to the carbon at δ_C_ 77.3 (C-12′), which is directly coupled to the signal at δ_H_ 3.97. The compound was identified as the new meroditerpenoid 2-[12-hydroxy-3,7,11,15-tetramethyl-2,6,10,14-hexadecatetraen-1-yl]-6-methyl-1,4-benzenediol, and attributed the trivial name paradoxhydroquinone (**5**). Unfortunately, **5** and **6** oxidized to **15** and **12** before any NOESY NMR experiments could be carried out. Therefore a bsNOESY NMR experiment was carried out on **15** and **12**, respectively, and the results related back to **5** and **6**. On biosynthetic grounds, the configuration of the double bonds was therefore confirmed as 2′*E*, 6′*E*, and 10′*E* for **5**, and 2′*E*, 6′*E*, and 10′*Z* for **6**.

**Table 3:** ^1^H and ^13^C NMR data (500 MHz, CDCl_3_) data for paradoxhydroquinone (**5**).

	Paradoxhydroquinone (5)			
Position	δC ^a^, Type	δH (*J* in Hz)	gCOSY	gHMBCAD
1	146.4, C			
2	127.6, C			
3	114.0, CH	6.46, d (2.5)	5, 1′	1, 4, 5, 1′
4	149.0, C			
5	115.5, CH	6.50, d (2.5)	3, 7	1, 3, 4, 7
6	125.5, C			
7	16.0, CH_3_	2.18, s	5	1, 5, 6
8				
1′	30.0, CH_2_	3.28, d (7.0)	3, 2′, 4′_w_, 18′_w_	1, 2, 3, 2′, 3′
2′	122.0, CH	5.26, t (7.0)	1′, 4′, 18′	1′, 4′, 18′
3′	138.1, C			
4′	39.5, CH_2_	2.08, m	1′_w_	5′, 18′
5′	26.1, CH_2_	2.13, m		4′, 7′
6′	124.4, CH	5.09, m		4′, 5′, 19′
7′	135.1, C			
8′	39.2, CH_2_	2.01, m		6′, 7′, 9′, 19′
9′	26.1, CH_2_	2.10, m	8′	7′, 10′, 11′
10′	126.2, CH	5.37, t (6.5)	9′, 20′	12′, 20′
11′	136.6, C			
12′	77.3, CH	3.97, dd (6.0,7.0)	13a′, 13b′	10′, 14′, 20′
13a′	34.2, CH_2_	2.20, m	12′, 16′, 17′	
13b′		2.28, ddd (7.0, 8.0, 14.0)	12′, 16′, 17′	12′, 14′, 15′
14′	120.2, CH	5.09, m	16′, 17′	
15′	134.7, C			
16′	18.0, CH_3_	1.63, s	14′	14′, 15′, 17′
17′	25.9, CH_3_	1.72, s	13b′, 14′	14′, 15′, 16′
18′	16.2, CH_3_	1.75, s	1′, 2′	2′, 3′, 4′
19′	16.1, CH_3_	1.59, s	5′, 6′	7′, 8′
20′	11.7, CH_3_	1.61, s	10′	10′, 11′, 12′
1-OH		ND ^b^		
4-OH		ND ^b^		
12′-OH		ND ^b^		

^a^ Carbon NMR assignments made on the basis of gHSQCAD and gHMBCAD NMR experiments. ^b^ Signal not detected.

Paradoxquinol (**10**), unlike many of the previously isolated meroditerpenoids, which show the presence of an AB aromatic system, only displayed a single aromatic proton NMR signal at δ 6.46, s (H-3), suggesting further substitution of the *p*-benzoquinone ring. The gHMBCAD NMR spectrum confirmed the structure of **10** as proposed by chemical profiling. The two deshielded methyl groups [δ_H_ 2.03 (s, H-7) and 2.00 (s, H-8)] showed correlations to carbons at δ_C_ 140.6 (C-5), 141.5 (C-6), and 187.7 (C-1), and δ 140.6 (C-5), 141.5 (C-6), and 187.9 (C-4), respectively (Table 4). Further analysis of the HMBC NMR data confirmed the position of attachment for the terminal and the secondary alcohol moieties as positions 20′ and 12′, respectively, and confirmed **10** as the new meroditerpenoid 5-[12*R*-hydroxy-11-(hydroxymethyl)-3,7,15-trimethyl-2,6,10,14-hexadecatetraen-1-yl]-2,3-dimethyl-1,4-benzoquinone, attributed the trivial name paradoxquinol (**10**). A single irradiation nOe NMR experiment confirmed the configuration of the double bond at position C-10′ as 10′*E* (irradiation of H-10′ showed a key enhancement to H-12′). Although this represents the first occurrence in a marine alga of a meroditerpenoid containing the position 8 methyl, this biosynthetic feature has been reported in terrestrial natural products [20,21]. It is suggested that the biosynthetic pathway of **10** is reminiscent to that of γ-tocotrienol (**16**).

Paradoxquinone (**15**) was isolated off-line as an inseparable mixture with 2-[11-(hydroxymethyl)-3,7,15-trimethyl-2,6,10,14-hexadecatetraen-1-yl]-6-methyl-1,4-benzoquinone (**12**) in a ratio of approximately 3:5. While complete NMR characterization was conducted on the mixture, the latter of these was identified as a separate entity via stop-flow HPLC-NMR. By analysis of the data obtained from both chemical profiling and off-line isolation, the latter of these compounds could be identified as the known meroditerpenoid 2-[11-(hydroxymethyl)-3,7,15-trimethyl-2,6,10,14-hexadecatetraen-1-yl]-6-methyl-1,4-benzoquinone (**12**). This compound had previously been reported as a derivative of a natural product; however, this represents the first report of its natural occurrence [17]. The ^1^H NMR spectrum of paradoxquinone (**15**) was similar to that of paradoxhydroquinone (**5**). Minor differences were observed for the aromatic protons (6.46, bs and 6.54, bs) and the terminal methyl group at position H-7 (δ_H_ 2.05, s) (Table 4). Complete analysis of the 2D NMR spectra resulted in the compound being identified as the new meroditerpenoid 2-[12-hydroxy-3,7,11,15-tetramethyl-2,6,10,14-hexadecatetraen-1-yl]-6-methyl-1,4-benzoquinone, attributed the trivial name paradoxquinone (**15**). For compound **12**, nOe enhancements were observed from each of the olefinic protons at positions H-2′, H-6′, and H-10′ to the methylene protons at H-4′, H-8′, and H-12′. For compound **15**, nOe enhancements were observed from the olefinic protons at positions H-2′ and H-6′ to the methylene protons at H-4′ and H-8′, together with an enhancement from the olefinic proton at H-10′ to the oxymethine proton at H-12′. The configuration of the double bonds was therefore confirmed as 2′*E*, 6′*E*, and 10′*E* for **15**, and 2′*E*, 6′*E*, and 10′*Z* for **12**.

The chirality of secondary allylic alcohols can be elucidated via the exciton chirality method (usually by preparation of a benzoate derivative) or by NMR spectroscopy of the diastereoisomeric Mosher or modified Mosher esters [22,23,24,25]. In the former methodology, the absolute configuration of acyclic secondary alcohols have been assigned by interpretation of the Cotton effects observed at two wavelength regions (~220 and ~240–260 nm, respectively) [22,24].

The CD spectra of fallahydroquinone (**1**), fallaquinone (**7**), and paradoxquinol (**10**) were recorded and Cotton effects observed in the circular dichroism spectra noted. The CD spectra of fallahydroquinone (**1**), fallaquinone (**7**), and paradoxquinol (**10**) all showed the presence of positive Cotton effects at 260, 247, and 245 nm, respectively, of Δε +2.0, +2.3, and +2.4 as well as at 224, 220, and 220 nm of Δε +1.8, +1.0, and +6.4, respectively. On the basis of the similarities in the CD spectra, these compounds were concluded to have the same (undefined) absolute configuration. Subsequent to these analyses, fallahydroquinone (**1**), fallaquinone (**7**), and paradoxquinol (**10**) completely degraded, which precluded any further attempts to address the absolute configuration by conversion to the corresponding Mosher esters.

**Table 4:** ^1^H and ^13^C NMR data (500 MHz, CDCl_3_) data for paradoxquinol (**10**) and paradoxquinone (**15**).

	Paradoxquinol (10)				Paradoxquinone (15)			
Position	δC ^a^, Type	δH (*J* in Hz)	gCOSY	gHMBCAD	δC ^a^, type	δH (*J* in Hz)	gCOSY	gHMBCAD
1	187.7, C				188.0, C			
2	148.1, C				148.5, C			
3	132.1, CH	6.46, s	1′		132.3, CH	6.46, bs	5, 1′	1, 4, 5
4	187.9, C				188.0, C			
5	140.6, C *				133.2, CH	6.54, bs	3, 7	1, 3, 4
6	141.5, C *				145.9, C			
7	12.4, CH_3_	2.03, s		1, 5, 6	16.0, CH_3_	2.05, s	5	1, 5, 6
8	12.1, CH_3_	2.00, s		4, 5, 6			3, 2′, 18′	1, 2, 3, 2′, 3′
1′	27.4, CH_2_	3.12, d (7.0)	3, 2′, 18′	1, 2, 3, 2′, 3′	27.5, CH_2_	3.13, d (7.5)	1′, 18	1′, 4′, 18′
2′	118.2, CH	5.15, t (7.0)	1′, 18′	4′	118.1, CH	5.15, t (7.5)		
3′	139.5, C				139.8, C			2′, 3′, 5′, 18′
4′	39.5, CH_2_	2.06, m		2′, 5′, 6′, 18′	39.6, CH_2_	2.07, m	6′, 18′_w_, 19′_w_	3′, 4′, 6′, 7′
5′	26.3, CH_2_	2.12, m	6′	4′, 6′	26.2, CH_2_	2.11, m	5′	4′, 5′, 19′
6′	124.4, CH	5.11, m	5′, 19′	4′, 8′	124.2, CH	5.10, m		
7′	134.7, C				135.1, C			6′, 7′, 10′
8′	39.5, CH_2_	2.06, m		19′	39.3, CH_2_	2.02, m	10′, 20′_w_	7′, 10′, 11′
9′	26.0, CH_2_	2.21, m	10′		26.2, CH_2_	2.11, m	9′, 20′	9′, 12′, 20′
10′	130.5, CH	5.53, t (7.0)	9′	12′, 20′	126.1, CH	5.38, t (7.0)		
11′	138.9, C				136.7, C		13a′	10′, 11′, 13′, 14′, 20′
12′	76.9, CH	4.16, dd (5.5, 7.5)	13a′, 13b′	10′	77.2, CH	3.97, dd (5.5, 7.5)	12′, 17′	
13a′	35.1, CH_2_	2.26, m	12′, 14′		34.2, CH_2_	2.20, m	12′, 17′	11′, 12′, 14′, 15′
13b′		2.43, ddd (7.5, 8.5, 14.5)	12′, 13a′, 14′	12′, 14′		2.27, m	17′	
14′	119.9, CH	5.11, m	16′, 17′		120.3, CH	5.09, m		
15′	135.4, C				134.6, C			14′, 15′
16′	18.1, CH_3_	1.65, s	13a′, 13b′	14′, 15′, 17′	18.0, CH_3_	1.63, s		14′, 15′, 16′
17′	25.9, CH_3_	1.73, s	13a′, 13b′, 14′	14′, 15′, 16′	25.9, CH_3_	1.72, s		2′, 3′
18′	16.1, CH_3_	1.62, s	1′, 2′	2′, 3′, 4′	16.1, CH_3_	1.61, s		7′
19′	16.1, CH_3_	1.60, s	5′, 6′	6′, 7′, 8′	16.1, CH_3_	1.61, s		10′, 11′
20′	58.5, CH_2_	4.26, d (3.5)		10′, 11′. 12′	11.7, CH_3_	1.61, s		
12′-OH		ND ^b^				ND ^b^		
20′-OH		ND ^b^						

^a^ Carbon NMR assignments made on the basis of gHSQCAD and gHMBCAD NMR experiments. ^b^ Signal not detected. * Indicates signals interchangeable

The compounds isolated from *S. paradoxum* in this study also occur in many other *Sargassum* species. However, two of the compounds—fallahydroquinone (**1**) and fallaquinone (**7**)—have only been reported from one other *Sargassum* species (*S. fallax*) [7]. On this basis, it is proposed that the presence of these two compounds could potentially be used as chemotaxonomic biomarkers for *S. paradoxum* and *S. fallax*.

### 2.3. Antimicrobial Studies

Fallahydroquinone (**1**) and fallaquinone (**7**) have been previously reported to display slight antitumor activity [7]. Sargahydroquinoic acid (**2**) and sargaquinoic acid (**11**) display similar antitumor [7,26] and peroxynitrite-scavenging activity [27], as well as antiplasmodial activity against malaria parasites [28]. Sargahydroquinoic acid (**2**) also shows insecticidal activity [29], selective vasodilatation effects [30], the ability to stimulate adipocyte differentiation [31], and radical scavenging activity [6]. Sargaquinoic acid (**11**) shows weak antimicrobial activity [7], the ability to promote neurite outgrowth [32], the ability to induce cell apoptosis [33], antioxidant activity [34], and cholinesterase inhibition activity [35]. Sargaquinal (**8**) displays antiplasmodial activity against malaria parasites [28]. A range of activities have been reported for sargaquinone (**13**) including radical scavenging [36], anti-inflammatory activity [37], activity against P-388 cells [7,38], and ichthyotoxicity to certain species of fish [39]. Antileishmanial activity has been reported for 9-(6-hydroxy-2,8-dimethyl-2*H*-1-benzopyran-2-yl)-6-methyl-2-(4-methyl-3-penten-1-yl)-2,6-nonadienal (**14**) [19].

Antimicrobial evaluation of the crude extracts, selected silica column fractions, and the isolated meroditerpenoids was carried out against five bacteria and one fungus. Eleven of the isolated meroditerpenoids (**1**–**2**, **5**–**7**, **9**–**13**, and **15**), together with a commercially available antibiotic and antifungal agent, were tested at 1 mg/mL, with all displaying varying degrees of zones of inhibition in the antimicrobial assays (Table 5). Compared to the antibiotic ampicillin, the isolated compounds were far less potent against *S. aureus* and *S. pyogenes*. However, compounds **2**, **7**, **8**, and **13** were more potent against *P. aeruginosa* than ampicillin. In recent years, concern has grown around *P. aeruginosa* with regards to its prevalence and multidrug resistance [40]; therefore, the importance of identifying possible drug leads against these bacteria remains highly relevant. There was no difference in activity between compounds with the hydroquinone or the *p*-benzoquinone moieties. The activity observed for sargaquinone (**13**), the simplest of the meroditerpenoids isolated, suggests that the unsubstituted meroditerpenoid skeleton is responsible for the activity against *P. aeruginosa*. The addition of an alcohol group at position 12′ or 20′ (**1**, **5**, **6**, **12**, and **15**) appears to reduce the activity against *P. aeruginosa*, but increases the activity against *S. pyogenes*. Finally, incorporation of a carboxylic acid at position C-20′ (**2** and **11**) gives rise to activity against *S. aureus* and *S. aureus* MRSA.

## 3. Experimental Section

### 3.1. General Experimental Procedures

All organic solvents used were analytical reagent (AR or GR), UV spectroscopic, or HPLC grades with milli-Q water also being used. Optical rotations were carried out using a 1.5-mL cell on a Rudolph Research Analytical Autopol IV automatic polarimeter, set to the Na 589 nm wavelength. UV/VIS spectra were recorded on an Agilent CARY 60 spectrophotometer, using ethanol. CD spectra were obtained on a Jasco 815 Circular Dichroism spectrometer in ethanol. FTIR spectra were recorded as a film using a NaCl disk on a Perkin-Elmer Spectrum One FTIR spectrometer. ^1^H (500 MHz), ^13^C (125 MHz), and 1D nOe spectra were acquired in CDCl_3_ on a 500 MHz Agilent DD2 NMR spectrometer with referencing to solvent signals (δ 7.26 and 77.0 ppm). Two-dimensional NMR experiments recorded included gradient correlation spectroscopy (gCOSY), heteronuclear single-quantum correlation spectroscopy with adiabatic pulses (HSQCAD), gradient heteronuclear multiple-bond spectroscopy with adiabatic pulses (gHMBCAD), and band selective 2D nuclear overhauser enhancement spectroscopy (bsNOESY) experiments. ESI mass spectra were obtained on a Micromass Platform II mass spectrometer equipped with a LC-10AD Shimadzu solvent delivery module (50% CH_3_CN/H_2_O at a flow rate of 0.2 mL/min) in both the positive and negative ionization modes using cone voltages between 20 and 30 V. Silica gel flash chromatography was carried out using Davisil LC35Å silica gel (40–60 mesh) with a 20% stepwise solvent elution from 100% petroleum spirits (60–80 °C) to 100% dichloromethane to 100% ethyl acetate and finally to 100% methanol. HPLC-NMR was carried out on a 500 MHz Agilent DD2 NMR spectrometer equipped with a Varian ^1^H[^13^C] pulsed field gradient flow probe with a 60-μL active volume flow cell coupled to a Varian Prostar 210 solvent delivery system, a Prostar 430 Autosampler, and a Prostar 335 PDA detector. The HPLC-NMR analyses were carried out using CORBA communication and operated with VnmrJ software. The 2H resonance observed from the D_2_O was used to obtain a field-frequency lock. The resonances from the HOD and the methyl of the acetonitrile were suppressed using the water enhanced through transverse gradients (WET) solvent suppression experiment [41]. The residual HOD resonance of D_2_O was referenced to 4.64 ppm. For both on-flow and stop-flow HPLC-NMR modes, 50-μL injections (4995 µg) of the dichloromethane extract were injected onto an Agilent Eclipse Plus C_18_ (150 × 4.6) 5-µ column using a solvent composition of 75% CH_3_CN/D_2_O at a flow rate of 1 mL/min. In the stop-flow HPLC-NMR mode WET1D, gCOSY, HSQCAD, gHMBCAD, and 1D nOe NMR experiments were acquired. HRESIMS was carried out on an Agilent 6200 Series TOF system (ESI operation conditions of 8 L/min N2, 325 °C drying gas temperature, and 3500 V capillary voltage) equipped with an Agilent 1200 Series LC solvent delivery module (100% CH_3_OH at a flow rate of 0.3 mL/min) in either the positive or negative ionization modes. The instrument was calibrated using the “Agilent Tuning Mix” with purine as the reference compound and the Hewlett–Packard standard HP0921. HRESILCMS was carried out on the same system and conditions using an Agilent Eclipse Plus C_18_ (4.6 × 150) 5-µ column using a solvent composition of 75% CH_3_CN/H_2_O at a flow rate of 1 mL/min. All analytical HPLC analyses and method development were performed on a Dionex P680 solvent delivery system equipped with a PDA100 UV detector (operated using “Chromeleon” software). Analytical HPLC analyses were carried out using either a gradient method 0–2 min 10% CH_3_CN/H_2_O; 14–24 min 75% CH_3_CN/H_2_O; 26–30 min 100% CH_3_CN; and 32–40 min 10% CH_3_CN/H_2_O or an isocratic method (either 100%, 95%, 90%, or 85% CH_3_CN/H_2_O) on an Alltech Alltima HP C_18_ (250 × 4.6) 5-µ column at a flow rate of 1.0 mL/min. Semi-preparative HPLC was carried out on a Varian Prostar 210 solvent delivery system equipped with a Prostar 335 PDA detector (operated using “Star Workstation” software) using an isocratic method (either 100%, 95%, 90%, or 85% CH_3_CN/H_2_O) and an Alltech Alltima C_18_ (250 × 10) 5-µ column at a flow rate of 3.5 mL/min.

### 3.2. Biological Evaluation

Crude extracts, enriched fractions, and compounds isolated from the marine alga, together with the standard antibiotic (ampicillin, Sigma-Aldrich, Castle Hill, Australia) and antifungal (carbendazim, Sigma-Aldrich, Castle Hill, Australia) compounds, were evaluated against six microorganisms (five bacteria and one fungus) at concentrations of 50, 25, or 1 mg/mL (see Table 5). Each microorganism was prepared by creating a 0.5 McFarlane solution suspension. Lawn cultures were prepared on either Mueller–Hinton or Brain Heart Infusion Agar (BHIA) (used for *S. pyogenes*). Then 20 µL of the crude extracts, enriched fractions, pure compounds or the standard compounds (ampicillin and carbendazim) were pipetted onto 6 mm diameter filter paper disks and their solvents evaporated. These disks were then placed onto the prepared lawn cultures and incubated at 37 °C overnight. Active antimicrobial samples displayed a zone of inhibition outside the disk, which was measured in mm as the radius of inhibition for each bacteria/fungi. The five test bacteria were *Eschericha coli* (ATCC 25922), *Staphylococcus aureus* (ATCC 25923), *Staphylococcus aureus* MRSA (344/2-32), *Pseudomonas aeruginosa* (ATCC 27853), and *Streptococcus pyogenes* (345/1). The test fungus was *Candida albicans* (ATCC 10231).

### 3.3. Marine Alga Material

The marine brown alga was collected by SCUBA at a depth of 3 m on 21 April 2010 from Governor Reef near Indented Head, Port Phillip Bay, Victoria, Australia and then stored at −80 °C. The alga was identified as *Sargassum paradoxum* (R. Brown ex Turner) Hooker and Harvey by Dr. Gerald Kraft (Honorary Principal Fellow), Faculty of Science, School of Botany, The University of Melbourne, Australia. A voucher specimen (designated the code number 2010–12) is deposited at the School of Applied Sciences (Discipline of Applied Chemistry), RMIT University.

### 3.4. Chemical Profiling

Chemical profiling was carried out on the dichloromethane soluble extract of the alga employing HPLC-NMR and HPLC-MS methodologies. Details of these analyses are provided in Section 3.1. The dichloromethane extract (77 mg) was dissolved in HPLC-NMR grade CH_3_CN (771 μL) and filtered through a 0.45 PTFE membrane filter (Grace Davison Discovery Sciences).

**Table 5:** Antimicrobial activity of the crude extract, selected silica column fractions, and pure compounds obtained from *S. paradoxum*, together with commercial standard antibiotic and antifungal compounds, showing zones of inhibition (mm).

	Microorganism	*E. coli*	*S. aureus*	*S. aureus MRSA*	*P. aeruginosa*	*S. pyogenes*	*C. albicans*
	Concentration (mg/mL)	ATCC 25922	ATCC 25923	344/2-32	ATCC 27853	345/1	ATCC 10231
Dichloromethane extract	50	ND ^g^	2	1	ND ^g^	5	ND ^g^
Methanol extract	50	ND ^g^	1	1	10	3	4
Silica fraction **1** ^a^	25	ND ^g^	ND ^g^	ND ^g^	ND ^g^	ND ^g^	ND ^g^
Silica fraction **4** ^b^	25	ND ^g^	ND ^g^	ND ^g^	ND ^g^	1	ND ^g^
Silica fraction **6** ^c^	50	ND ^g^	ND ^g^	1	ND ^g^	1	ND ^g^
Silica fraction **10** ^d^	25	ND ^g^	ND ^g^	1	4	3	ND ^g^
Silica fraction **13** ^e^	50	ND ^g^	ND ^g^	1	ND ^g^	3	ND ^g^
Silica fraction **17** ^f^	50	ND ^g^	ND ^g^	ND ^g^	ND ^g^	ND ^g^	ND ^g^
Fallahydroquinone (**1**)	1	ND ^g^	ND ^g^	ND ^g^	ND ^g^	1	ND ^g^
Sargahydroquinoic acid (**2**)	1	ND ^g^	1	1	2	3	ND ^g^
Paradoxhydroquinone (**5**) & compound (**6**)	1	ND ^g^	ND ^g^	ND ^g^	ND ^g^	3	ND ^g^
Fallaquinone (**7**)	1	ND ^g^	ND ^g^	ND ^g^	4	ND ^g^	ND ^g^
Sargaquinal (**8**)	1	ND ^g^	ND ^g^	ND ^g^	3	1	ND ^g^
Paradoxquinol (**10**)	1	ND ^g^	ND ^g^	ND ^g^	ND ^g^	1	ND ^g^
Sargaquinoic acid (**11**)	1	ND ^g^	1	1	1	3	ND ^g^
Paradoxquinone (**15**) & compound (**12**)	1	ND ^g^	ND ^g^	ND ^g^	ND ^g^	2	ND ^g^
Sargaquinone (**13**)	1	ND ^g^	ND ^g^	ND ^g^	5	ND ^g^	ND ^g^
Ampicillin (antibiotic)	1	ND ^g^	15	3	2	20	NT ^h^
Carbendazim (antifungal)	1	NT ^h^	NT ^h^	NT ^h^	NT ^h^	NT ^h^	ND ^g^

^a^ 100% to 80% petroleum spirits (60–80 °C)/dichloromethane; ^b^ 20% petroleum spirits (60–80 °C)/dichloromethane; ^c^ 80% dichloromethane/ethyl acetate; ^d^ 20% dichloromethane/ethyl acetate; ^e^ 80% ethyl acetate/methanol; ^f^ 100% methanol; ^g^ Indicates no zone of inhibition detected; ^h^ Indicates not tested.

### 3.5. Extraction and Isolation

The frozen marine alga (88 g, wet weight) was extracted with 3:1 methanol/dichloromethane (2 L). The crude extract was then decanted and concentrated under reduced pressure and sequentially solvent partitioned (triturated) into dichloromethane- and methanol-soluble extracts, respectively. Approximately 450 mg of the dichloromethane extract was subjected to flash silica gel column chromatography (20% stepwise elution from petroleum spirits (60–80 °C) to dichloromethane to ethyl acetate and finally to methanol). The 40% petroleum spirits/dichloromethane silica gel column fraction was subjected to reversed phase HPLC (100% CH_3_CN) to yield sargaquinone (**13**) (2.5 mg, 0.02%). The 80% dichloromethane/ethyl acetate silica gel fraction was subjected to reversed-phase HPLC (95% CH_3_CN/H_2_O) to yield paradoxquinone (**15**) and 2-[11-(hydroxymethyl)-3,7,15-trimethyl-2,6,10,14-hexadecatetraen-1-yl]-6-methyl-1,4-benzoquinone (**12**) as an inseparable mixture (6.0 mg, 0.04%), and sargaquinal (**8**) (3.0 mg, 0.02%). The 40% dichloromethane/ethyl acetate to 60% dichloromethane/ethyl acetate silica gel column fractions were combined and subjected to Sephadex LH-20 size exclusion column chromatography using 100% methanol. The third fraction resulting from this analysis was subjected to reversed phase HPLC (85% CH_3_CN/H_2_O) to yield fallaquinone (**7**) (1.3 mg, 0.01%) and paradoxquinol (**10**) (0.2 mg, 0.001%). The remaining half of the dichloromethane extract was purified separately using reversed-phase HPLC (90% CH_3_CN/H_2_O) to yield fallahydroquinone (**1**) (7.3 mg, 0.05%), sargahydroquinoic acid (**2**) (17.6 mg, 0.11%), paradoxhydroquinone (**5**) and 2-[11-(hydroxymethyl)-3,7,15-trimethyl-2,6,10,14-hexadecatetraen-1-yl]-6-methyl-1,4-benzenediol (**6**) as an inseparable mixture (6.5 mg, 0.04%), sargaquinoic acid (**11**) (13.1 mg, 0.08%), and 9-(6-hydroxy-2,8-dimethyl-2*H*-1-benzopyran-2-yl)-6-methyl-2-(4-methyl-3-penten-1-yl)-2,6-nonadienal (**14**) (0.4 mg, 0.005%). The percentage yields are reported on the basis of the dry mass of the alga extracted (refer to the supporting information for details of the bioassay-guided isolation scheme adopted).

### 3.6. On-Line (HPLC-NMR & HPLC-MS) Characterization of Compounds

2-[12*S*-Hydroxy-11-(hydroxymethyl)-3,7,15-trimethyl-2,6,10,14-hexadecatetraen-1-yl]-6-methyl-1,4-benzenediol (fallahydroquinone) (**1**): HPLC-NMR WET1D NMR (500 MHz, 75% CH_3_CN/D_2_O, suppression of HDO and CH_3_CN at t δ_H_ 4.64 and 2.82 ppm, respectively) obtained from stop-flow mode (see Table 1); HRESILCMS *m*/*z* 427.2846 (calcd for C_27_H_39_O_4_, 427.2849).

12-(2,5-Dihydroxy-3-methylphenyl)-6,10-dimethyl-2-(4-methyl-3-penten-1-yl)-(2*Z*,6*E*,10*E*)-2,6,10-dodecatrienoic acid (sargahydroquinoic acid) (**2**): HPLC-NMR WET1D NMR (500 MHz, 75% CH_3_CN/D_2_O, suppression of HDO and CH_3_CN at t δ_H_ 4.64 and 2.82 ppm respectively) obtained from stop-flow mode (see Table 1); HRESILCMS *m*/*z* 425.2694 (calcd for C_27_H_37_O_4_, 425.2692).

2-[12-Hydroxy-3,7,11,15-tetramethyl-2,6,10,14-hexadecatetraen-1-yl]-6-methyl-1,4-benzenediol (paradoxhydroquinone) (**5**): HPLC-NMR WET1D NMR (500 MHz, 75% CH_3_CN/D_2_O, suppression of HDO and CH_3_CN at t δ_H_ 4.64 and 2.82 ppm, respectively) obtained from stop-flow mode δ 7.26 (1H, d, *J* = 3.0, H-5), 7.22 (1H, d, *J* = 3.0, H-3), 6.11 (1H, t, *J* = 7.0, H-2′), 6.06 (1H, t, *J* = 7.0, H-10′), 5.96 (2H, m, H-6′/H-14′), 4.06 (2H, d, *J* = 7.0, H-1′), 3.30 (1H, dd, *J* = 7.0, 7.0, H-12′), 2.95–3.04 (m), 2.96 (3H, s, H-7), 2.94 (2H, m, H-5′/H-13′*), 2.53 (3H, s, H-18′), 2.49 (3H, s, H-17′), 2.42 (3H, s, H-16′), 2.41 (3H, s, H-20′*), 2.37 (3H, s, H-19′*), ND (1-OH, 4-OH, 12′-OH) *signals interchangeable; HRESILCMS *m*/*z* 411.2899 (calcd for C_27_H_39_O_3_, 411.2899).

2-[11-(Hydroxymethyl)-3,7,15-trimethyl-2,6,10,14-hexadecatetraen-1-yl]-6-methyl-1,4-benzenediol (**6**): HPLC-NMR WET1D NMR (500 MHz, 75% CH_3_CN/D_2_O, suppression of HDO and CH_3_CN at t δ_H_ 4.64 and 2.82 ppm, respectively) obtained from stop-flow mode δ 7.25 (1H, d, *J* = 3.0, H-5), 7.22 (1H, d, *J* = 3.0, H-3), 6.10 (1H, t, *J* = 7.0, H-2′), 6.06 (1H, t, *J* = 7.5, H-10′), 5.95 (2H, m, H-6′/H-14′), 4.84 (2H, s, H-20′), 4.05 (2H, d, *J* = 7.0, H-1′), 2.95–3.04 (m), 2.97 (3H, s, H-7), 2.95 (2H, m, H-9′), 2.93 (2H, m, H-5′/H-13′*), 2.52 (3H, s, H-18′), 2.48 (3H, s, H-17′), 2.41 (6H, s, H-16′/H19′) ND (1-OH, 4-OH, 20′-OH) *signals interchangeable; HRESILCMS *m*/*z* 411.2899 (calcd for C_27_H_39_O_3_, 411.2899).

5-[12*S*-Hydroxy-11-(hydroxymethyl)-3,7,15-trimethyl-2,6,10,14-hexadecatetraen-1-yl]-6-methyl-1,4-benzoquinone (fallaquinone) (**7**): HPLC-NMR WET1D NMR (500 MHz, 75% CH_3_CN/D_2_O, suppression of HDO and CH_3_CN at t δ_H_ 4.64 and 2.82 ppm, respectively) obtained from stop-flow mode δ 7.42 (1H, bs, H-5), 7.28 (1H, bs, H-3), 6.30 (1H, t, *J* = 7.5, H-10′), 6.00 (1H, t, *J* = 7.0, H-2′), 5.95 (1H, t, *J* = 6.5, H-6′), 5.91 (1H, t, *J* = 6.5, H-14′), 4.95 (1H, d, *J* = 12.0, H-20a′), 4.89 (1H, d, *J* = 12.5, H-20b′), 4.87 (1H, dd, *J* = 6.0, 7.0, H-12′), 3.93 (2H, d, *J* = 7.0, H-1′), 2.95–3.10 (m), 3.08 (2H, m, H-13′), 3.03 (2H, m, H-9′), 2.96 (2H, m, H-5′), 2.50 (3H, s, H-17′), 2.46 (3H, s, H-18′), 2.42 (6H, s, H-16′/H-19′) ND (12′-OH, 20′-OH); HRESILCMS *m*/*z* 425.2694 (calcd for C_27_H_37_O_4_, 425.2692).

6,10-dimethyl-12-(5-methyl-3,6-dihydroxy-1,4-cyclohexadien-1-yl)-2-(4-methyl-3-pentenyl)-(2*E*,6*E*,10*E*)-2,6,10-dodecatrienal (sargahydroquinal) (**9**): UV (75% CH_3_CN/D_2_O) λ_max_ 238, 292; HPLC-NMR WET1D NMR (500 MHz, 75% CH_3_CN/D_2_O, suppression of HDO and CH_3_CN at t δ_H_ 4.64 and 2.82 ppm, respectively) obtained from stop-flow mode (see Table 2); HRESILCMS *m*/*z* 409.2745 (calcd for C_27_H_37_O_3_, 409.2743).

5-[12*S*-Hydroxy-11-(hydroxymethyl)-3,7,15-trimethyl-2,6,10,14-hexadecatetraen-1-yl]-2,3-dimethyl-1,4-benzoquinone (paradoxquinol) (**10**): HPLC-NMR WET1D NMR (500 MHz, 75% CH_3_CN/D_2_O, suppression of HDO and CH_3_CN at t δ_H_ 4.64 and 2.82 ppm, respectively) obtained from stop-flow mode δ 7.27 (1H, s, H-3), 6.29 (1H, t, *J* = 7.0, H-10′), 5.99 (1H, m, H-2′), 5.92 (2H, m, H-6′/H-14′), 4.95 (1H, d, *J* = 12.0, H-20a′), 4.88 (1H, d, *J* = 13.0, H-20b′), 4.87 (1H, dd, *J* = 6.0, 7.5, H-12′), 3.92 (2H, d, *J* = 7.5, H-1′), 2.95-3.10 (m), 2.49 (3H, s, H-18′), 2.45 (3H, s, H-17′), 2.41 (6H, s, H-16′/H-19′) ND (12′-OH, 20′-OH); HRESILCMS *m*/*z* 439.2850 (calcd for C_28_H_39_O_4_, 439.2849).

6,10-Dimethyl-12-(5-methyl-3,6-dioxo-1,4-cyclohexadien-1-yl)-2-(4-methyl-3-pentenyl)-(2*Z*,6*E*,10*E*)-2,6,10-dodecatrienoic acid (sargaquinoic acid) (**11**): HPLC-NMR WET1D NMR (500 MHz, 75% CH_3_CN/D_2_O, suppression of HDO and CH_3_CN at t δ_H_ 4.64 and 2.82 ppm, respectively) obtained from stop-flow mode δ 7.42 (1H, d, *J* = 1.5, H-5), 7.28 (1H, d, *J* = 1.5, H-3), 6.69 (1H, t, *J* = 7.5, H-10′), 6.00 (1H, t, *J* = 7.0, H-2′), 5.95 (1H, t, *J* = 6.5, H-6′), 5.91 (1H, t, *J* = 6.5, H-14′), 3.93 (2H, d, *J* = 7.0, H1′), 3.31 (2H, dt, *J* = 7.5, 8.0, H-9′), 3.02 (2H, m, H-12′), 2.95–3.10 (m), 2.97 (2H, m, H-5′), 2.49 (3H, s, H-17′), 2.46 (3H, s, H-18′), 2.41 (3H, s, H-19′), 2.40 (3H, s, H-16′) ND (20′-OH); HRESILCMS *m*/*z* 423.2542 (calcd for C_27_H_35_O_4_, 423.2536).

2-[11-(Hydroxymethyl)-3,7,15-trimethyl-2,6,10,14-hexadecatetraen-1-yl]-6-methyl-1,4-benzoquinone (**12**): HPLC-NMR WET1D NMR (500 MHz, 75% CH_3_CN/D_2_O, suppression of HDO and CH_3_CN at t δ_H_ 4.64 and 2.82 ppm, respectively) obtained from stop-flow mode δ 7.41 (1H, bs, H-5), 7.27 (1H, bs, H-3), 6.05 (1H, t, *J* = 7.5, H-10′), 6.00 (1H, t, *J* = 7.5, H-2′), 5.94 (2H, m, H-6′/H-14′), 4.84 (2H, s, H-20′), 3.93 (2H, d, *J* = 7.5, H1′), 2.95–3.10 (m), 2.49 (3H, s, H-17′), 2.46 (3H, s, H-18′), 2.41 (6H, s, H-16′/H-19′) ND (20′-OH); HRESILCMS *m*/*z* 409.2742 (calcd for C_27_H_37_O_3_, 409.2743).

### 3.7. Off-Line Characterization of Compounds

(2′*E*,6′*E*,10′*Z*)-2-[12*S*-Hydroxy-11-(hydroxymethyl)-3,7,15-trimethyl-2,6,10,14-hexadecatetraen-1-yl]-6-methyl-1,4-benzenediol (fallahydroquinone) (**1**): unstable yellow oil; ${\left[\text{α}\right]}_{D}^{25}$ +45.1 (*c* 0.035, CHCl_3_); CD (EtOH) λ_max_ (Δε) 202 (+6.1), 221 (+1.6), 224 (1.8), 243 (−0.2), 260 (+2.0), 275 (+0.5), 290 (+0.6); UV (EtOH) λ_max_ (log ε) 252 (3.60), 288 (3.58); IR ν_max_ 3391, 2925, 2855, 1652, 1607, 1470, 1381, 1197, 1037 cm^−1^. All off-line NMR spectroscopic and mass spectrometric data were identical to those reported in the literature; however, some carbon and proton NMR chemical shift reassignments have been made [7].

(2′*E*,6′*E*,10′*Z*)-12-(2,5-Dihydroxy-3-methylphenyl)-6,10-dimethyl-2-(4-methyl-3-penten-1-yl)-2,6,10-dodecatrienoic acid (sargahydroquinoic acid) (**2**): yellow oil which darkened with time. All off-line NMR spectroscopic and mass spectrometric data were identical to those reported in the literature [7,8].

(2′*E*,6′*E*,10′*Z*)-2-[12-Hydroxy-3,7,11,15-tetramethyl-2,6,10,14-hexadecatetraen-1-yl]-6-methyl-1,4-benzenediol (paradoxhydroquinone) (**5**): dark yellow oil; ^1^H and ^13^C NMR (500 MHz, CDCl_3_) (see Table 3); ESIMS *m*/*z* 411 [M − H]^−^; HRESIMS *m*/*z* 411.2898 (calcd for C_27_H_39_O_3_, 411.2899).

(2′*E*,6′*E*,10′*Z*)-2-[11-(Hydroxymethyl)-3,7,15-trimethyl-2,6,10,14-hexadecatetraen-1-yl]-6-methyl-1,4-benzenediol (**6**): dark yellow oil. All off-line NMR spectroscopic and mass spectrometric data were identical to those reported in the literature [16]. Acquisition of the 1D and 2D NMR data has resulted in the first complete assignment of this compound and is provided in the supporting information.

(2′*E*,6′*E*,10′*Z*)-5-[12*S*-Hydroxy-11-(hydroxymethyl)-3,7,15-trimethyl-2,6,10,14-hexadecatetraen-1-yl]-6-methyl-1,4-benzoquinone (fallaquinone) (**7**): unstable yellow oil which darkened with time; ${\left[\text{α}\right]}_{D}^{25}$ +31.4 (*c* 0.065, CHCl_3_); CD (EtOH) λ_max_ (Δε) 206 (+1.8), 220 (+1.0), 238 (+2.1), 247 (+2.3), 262 (+1.6), 270 (+1.9), 286 (+0.1), 294 (+0.3); UV (EtOH) λ_max_ (log ε) 254 (4.10); IR ν_max_ 3392, 2924, 2855, 1652, 1614, 1510, 1436, 1378, 1295, 1195, 1156 cm^−1^. All off-line NMR spectroscopic and mass spectrometric data were identical to those reported in the literature [7].

(2′*E*,6′*E*,10′*Z*)-6,10-Dimethyl-12-(5-methyl-3,6-dioxo-1,4-cyclohexadien-1-yl)-2-(4-methyl-3-pentenyl)-2,6,10-dodecatrienal (sargaquinal) (**8**): yellow oil which darkened with time; ^1^H NMR (500 MHz, CDCl_3_) δ 9.34 (1H, s, H-20′), 6.54 (1H, bs, H-5), 6.45 (1H, bs, H-3), 6.43 (1H, t, *J* = 7.5, H-10′), 5.15 (2H, t, *J* = 7.5, H-2′, H-6′), 5.09 (1H, m, H-14′), 3.12 (2H, d, *J* = 7.5, H1′), 2.45 (2H, dt, *J* = 7.0, 7.5, H-9′), 2.26 (2H, t, *J* = 7.5 Hz, H-12′), 2.17 (2H, m, H-8′), 2.14 (2H, m, H-5′), 2.07 (2H, m, H-4′), 2.06 (3H, s, H-7), 2.03 (2H, m, H-13′), 1.66 (3H, s, H-16′), 1.63 (3H, s, H-18′), 1.62 (3H, s, H-19′), 1.57 (3H, s, H-17′); ^13^C NMR (125 MHz, CDCl_3_) δ 195.2 (CH, C-20′), 188.0 (C, C-1, C-4), 155.0 (CH, C-10′), 148.4 (C, C-2), 146.0 (C, C-6), 143.2 (C, C-11′), 139.7 (C, C-3′), 133.9 (C, C-7′), 133.2 (CH, C-5), 132.4 (C, C-15′), 132.2 (CH, C-3), 125.1 (CH, C-6′), 123.6 (CH, C-14′), 118.2 (CH, C-2′), 39.5 (CH_2_, C-4′), 38.3 (CH_2_, C-8′), 27.5 (CH_2_, C-1′), 27.4 (CH_2_, C-9′), 27.0 (CH_2_, C-13′), 26.4 (CH_2_, C-5′), 25.7 (CH_3_, C-16′), 24.3 (CH_2_, C-12′), 17.7 (CH_3_, C-17′), 16.0 (CH_3_, C-7, C-18′, C-19′).

(2′*E*,6′*E*,10′*Z*)-5-[12*S*-Hydroxy-11-(hydroxymethyl)-3,7,15-trimethyl-2,6,10,14-hexadecatetraen-1-yl]-2,3-dimethyl-1,4-benzoquinone (paradoxquinol) (**10**): unstable yellow oil which darkened with time; ${\left[\text{α}\right]}_{D}^{25}$ +48.5 (*c* 0.0125, CHCl_3_); CD (EtOH) λ_max_ (Δε) 218 (+6.2), 220 (+6.4), 235 (−4.2), 245 (+2.4), 255 (+1.2); UV (EtOH) λ_max_ (log ε) 232 (4.17), 254 (4.32); IR ν_max_ 3401, 2924, 2853, 1652, 1511, 1456, 1249 cm^−1^; ^1^H and ^13^C NMR (500 MHz, CDCl_3_) (see Table 4); ESIMS *m*/*z* 439 [M − H]^−^; HRESIMS *m*/*z* 439.2850 (calcd for C_28_H_39_O_4_, 439.2849).

(2′*E*,6′*E*,10′*Z*)-6,10-Dimethyl-12-(5-methyl-3,6-dioxo-1,4-cyclohexadien-1-yl)-2-(4-methyl-3-pentenyl)-2,6,10-dodecatrienoic acid (sargaquinoic acid) (**11**): yellow oil which darkened with time. All off-line NMR spectroscopic and mass spectrometric data were identical to those reported in the literature [7,17].

(2′*E*,6′*E*,10′*Z*)-2-[11-(Hydroxymethyl)-3,7,15-trimethyl-2,6,10,14-hexadecatetraen-1-yl]-6-methyl-1,4-benzoquinone (**12**): dark yellow oil. All off-line NMR spectroscopic and mass spectrometric data were identical to those reported in the literature [16,17]. Acquisition of the 1D and 2D NMR data has resulted in the first complete assignment of this compound and is provided in the supporting information.

(2′*E*,6′*E*,10′*Z*)-2-Methyl-6-(3,7,11,15-tetramethyl-2,6,10,14-hexadecatetraenyl)-1,4-benzoquinone (sargaquinone) (**13**): dark yellow oil. All off-line NMR spectroscopic and mass spectrometric data were identical to those reported in the literature; however, some carbon NMR chemical shift reassignments have been made [18].

(3′*E*,7′*Z*)-9-(6-Hydroxy-2,8-dimethyl-2*H*-1-benzopyran-2-yl)-6-methyl-2-(4-methyl-3-penten-1-yl)-2,6-nonadienal (**14**): dark yellow oil; UV (90% CH_3_CN/H_2_O) λ_max_ 232, 273s, 334; IR ν_max_ 3351, 2961, 2925, 2853, 2718, 1725, 1684, 1640, 1610, 1508, 1456, 1376, 1250 cm^−1^. All off-line NMR spectroscopic and mass spectrometric data were identical to those reported in the literature [19].

(2′*E*,6′*E*,10′*Z*)-2-[12-Hydroxy-3,7,11,15-tetramethyl-2,6,10,14-hexadecatetraen-1-yl]-6-methyl-1,4-benzoquinone (paradoxquinone) (**15**): yellow oil which darkened with time; ^1^H and ^13^C NMR (500 MHz, CDCl_3_) (see Table 4); ESIMS *m/z* 409 [M − H]^−^; HRESIMS *m*/*z* 409.2755 (calcd for C_27_H_37_O_3_, 409.2743).

## 4. Conclusions

The study of the marine brown alga *S. paradoxum* successfully implemented the use of HPLC-NMR and HPLC-MS, in combination with the use of databases, to rapidly dereplicate, identify, and deduce the structures of nine meroditerpenoids (**1**, **2**, **5**–**7**, and **9**–**12**). In particular, chemical profiling was an invaluable tool for the on-line identification of sargahydroquinal (**9**), which rapidly degraded when an off-line bioassay-guided isolation was employed, as well as for the meroditerpenoids **5**, **6**, and **12**, which could be resolved and identified as individual components via stop-flow HPLC-NMR. This study reports, for the first time, the complete 1D and 2D NMR characterization and identification of two known (**1** and **2**) natural products via stop-flow HPLC-NMR and HPLC-MS. The bioassay-guided isolation approach confirmed the identity of the compounds responsible for the dichloromethane crude extract antimicrobial activity. Subsequent biological evaluation of the isolated meroditerpenoids indicated that there were no significant differences in antimicrobial activity for 11 of the meroditerpenoids isolated. Off-line bioassay-guided isolation also permitted the first complete NMR characterization of 2-[11-(hydroxymethyl)-3,7,15-trimethyl-2,6,10,14-hexadecatetraen-1-yl]-6-methyl-1,4-benzenediol (**6**) and 2-[11-(hydroxymethyl)-3,7,15-trimethyl-2,6,10,14-hexadecatetraen-1-yl]-6-methyl-1,4-benzoquinone (**12**) to be carried out. It is proposed that the presence of fallahydroquinone (**1**) and fallaquinone (**7**) could be used as potential chemotaxonomic markers for both *S*. *paradoxum* and *S*. *fallax*, which have only been reported from these two species of *Sargassum*.

## Supplementary Information

Complete HPLC-NMR and HPLC-MS analysis profiles of the crude extract of *S. paradoxum* are included. NMR spectroscopic and mass spectrometric data obtained from the HPLC-NMR and HPLC-MS analyses have been provided for the meroditerpenoids (**1**, **2**, **5**–**7**, and **9**–**12**). The off-line NMR spectroscopic and mass spectrometric data obtained for the new meroditerpenoids (**5**, **10**, and **15**) and for compounds **6** and **12**, which have been characterized completely for the first time, have also been made available. This material is available free of charge via the Internet at http://pubs.acs.org.
